# A Novel Therapeutic Target, BACH1, Regulates Cancer Metabolism

**DOI:** 10.3390/cells10030634

**Published:** 2021-03-12

**Authors:** Joselyn Padilla, Jiyoung Lee

**Affiliations:** Department of Biochemistry and Molecular Medicine, School of Medicine & Health Sciences, George Washington University, Washington, DC 20037, USA; joselynp@gwu.edu

**Keywords:** BTB and CNC homology 1 BACH1, mitochondrial metabolism, glycolysis, heme oxygenase 1 (HMOX1), mitochondrial electron transport chain (ETC), Nrf2 (encoded by Nfe2l2), metformin, hemin, breast cancer, lung cancer

## Abstract

BTB domain and CNC homology 1 (BACH1) is a transcription factor that is highly expressed in tumors including breast and lung, relative to their non-tumor tissues. BACH1 is known to regulate multiple physiological processes including heme homeostasis, oxidative stress response, senescence, cell cycle, and mitosis. In a tumor, BACH1 promotes invasion and metastasis of cancer cells, and the expression of BACH1 presents a poor outcome for cancer patients including breast and lung cancer patients. Recent studies identified novel functional roles of BACH1 in the regulation of metabolic pathways in cancer cells. BACH1 inhibits mitochondrial metabolism through transcriptional suppression of mitochondrial membrane genes. In addition, BACH1 suppresses activity of pyruvate dehydrogenase (PDH), a key enzyme that converts pyruvate to acetyl-CoA for the citric acid (TCA) cycle through transcriptional activation of pyruvate dehydrogenase kinase (PDK). Moreover, BACH1 increases glucose uptake and lactate secretion through the expression of metabolic enzymes involved such as hexokinase 2 (HK2) and glyceraldehyde 3-phosphate dehydrogenase (GAPDH) for aerobic glycolysis. Pharmacological or genetic inhibition of BACH1 could reprogram by increasing mitochondrial metabolism, subsequently rendering metabolic vulnerability of cancer cells against mitochondrial respiratory inhibition. Furthermore, inhibition of BACH1 decreased antioxidant-induced glycolysis rates as well as reduced migration and invasion of cancer cells, suggesting BACH1 as a potentially useful cancer therapeutic target.

## 1. Introduction

Metabolism is an essential process to acquire necessary nutrients from outside of cells and utilize them into biomass to maintain cell viability. In a tumor microenvironment that has limited nutrients and oxygen, nutrient availability and nutritional stresses often rewire metabolic pathways of cancer cells. Hence, cancer cells exhibit distinct metabolic phenotypes to support their survival and proliferation states in tumor microenvironment [1]. The most common metabolic phenotypic changes in cancer cells are increased glucose uptake for aerobic glycolysis and lactate production even in the presence of oxygen [2]. In response to the metabolic stress or oncogenic signals, cancer cells also utilize alternative carbon and energy sources such as fatty acids and amino acids, including glutamine, using macropinocytosis to fulfill increased energy demands [3,4,5,6]. Due to the distinct cancer metabolism, blocking primary metabolic pathways of cancer cells could effectively reduce proliferation of cancer cells or inhibit survival of cancer cells, generating a cancer vulnerability. Currently, several metabolic drugs targeting altered and activated metabolic pathways of cancer cells have been approved, or are under current clinical trials as a potential cancer treatment [7]. However, inhibition of particular metabolic pathways can be ineffective or less effective, because cancer cells have the metabolic flexibility to use different substrates or alternative metabolic pathways to adapt various resources for their survival [8,9]. Thus, rewiring the primary cancer metabolism is required to drive cancer cells more targetable and susceptible. To this end, it is necessary to understand regulatory mechanisms of cancer metabolism that can contribute to cancer therapeutics, as well as, prognostic and predictive biomarkers for cancer patients. Recent several studies identified a new functional role of BACH1 in cancer metabolism regulation. BACH1 works as a metabolic driver in response to the intracellular and extracellular signals in breast and lung cancer cells [10,11,12]. This review highlights recent advances in an understanding of the newly discovered essential role of BACH1 on cancer metabolism and its potential as a therapeutic target for cancer therapy. Although BACH1 plays critical roles for other physiological processes, including cellular senescence independent of p53, spindle orientation rearrangement during mitosis upon phosphorylation on BACH1, and myocardial function against stress; these are not discussed in this review [13,14,15,16].

## 2. Heme-Binding Transcription Factor, BACH1

The BTB domain and CNC homology (BACH), is a member of the Cap ‘n’ Collar (CNC)/pox virus and zinc finger (POZ) protein that contains a basic leucine zipper (bZIP) domain and a Bric-a-brac-Tramtrack-Broad complex (BTB) domain at the N-terminal region [17]. Two BACH family protein members, BACH1 and BACH2, have homologous sequences and structures containing a BTB and the bZIP domain, heme-binding motifs, and the cytoplasmic localization signal (CLS) [17]. Both BACH1 and BACH2 are widely expressed in most human tissues. In particular, BACH1 mRNA is abundantly enriched in cell types such as neutrophils, NK cells, monocytes, macrophages, and dendritic cells, while BACH2 mRNA in B cells and T cells, which are implying non-redundant functions of BACH1 and BACH2 [18]. As a member of the bZIP transcription factor family, the bZIP domain allows BACH1 to recognize the DNA elements known as MAF recognition elements (MAREs), which is well conserved both in human and mouse, forming a well characterized heterodimer with small MAF proteins including MAFF, MAFG, and MAFK [13,19]. Moreover, the BACH proteins are able to form a dimer (either homodimer or heterodimer) with other transcription factors, which give BACH1 proteins regulatory agility for transcription activity [20].

**Figure 1:**
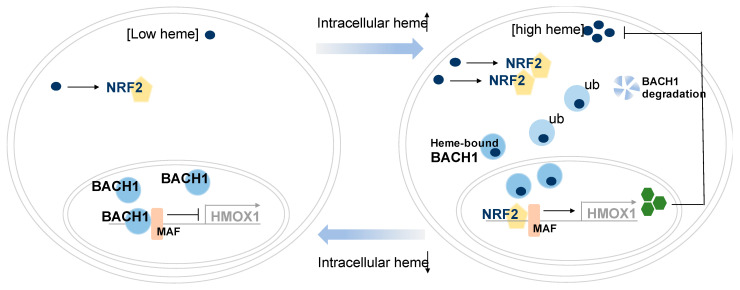
BACH1 regulates gene expression involved in heme homeostasis. Heme homeostasis regulation by BACH1 in cancer and non-cancerous cells are shown. BACH1 and NRF2 interplay for intercellular heme homeostasis. Low cellular heme levels enable BACH1 proteins to form a heterodimer with MAF proteins for transcriptional suppression of heme oxygenase 1 (HMOX1). Upon increasement of free heme levels that are synthesized in mitochondria or by extracellular treatment, activated NRF2 partners with MAF proteins for transactivation of *HMOX1*. Heme binds to BACH1 and heme-bound BACH1 undergoes nuclear export and further ubiquitin-dependent degradation. Expressed HMOX1 then degrades extra heme to maintain heme levels in cells. (Abbreviations: BTB and CNC Homology 1; BACH1, NRF2; NFE2L2, ub; Polyubiquitination.)

Another feature of BACH proteins is the multiple heme-binding cysteine-proline (CP) motifs that are located in 6 different sites at the C-terminal regions in BACH1, and in 5 different sites in BACH2 [15,18]. Heme is a compound of porphyrin class that contains an iron in the middle of the structure. Heme plays important regulatory roles in cells and provides a prosthetic group for many heme proteins, including hemoglobin and cytochrome c oxidase [21]. Biochemical assays using a mutation on cysteine (C) and proline (P) residues in BACH1 showed that BACH1 protein directly interacts with free heme. Heme-bound BACH1 proteins undergo proteasomal degradation after nuclear export. As a heme-binding transcription factor, BACH1 was revealed to sense intracellular heme levels to regulate heme homeostasis [22]. Glutathione-S-transferase (GST)-fused BACH1 derivatives showed heme affinity in vitro and the mutation of CP motifs in BACH1-interrupted heme interactions. Upon heme binding, BACH1 is released from the DNA and undergoes nuclear export for ubiquitin-dependent degradation, thus releasing transcription suppression of the BACH1 target gene, heme oxygenase 1 (*HMOX1* or *HO-1*) [23,24]. Simultaneously, accumulated Nrf2 (encoded by *NFE2L2*) replaces BACH1 to form a heterodimer with MAF proteins for transcriptional activation of target gene, *HMOX1* (Figure 1). Heme-induced and BACH1-mediated HMOX1 further catabolizes heme into biliverdin and CO and releases Fe^2+^ [21]. Consequently, BACH1 controls heme levels by regulating expression of HMOX1, generating a negative feedback loop.

As a transcriptional factor, genome-wide BACH1 bindings were validated using a chromatin immunoprecipitation (ChIP) combined with deep-sequencing (ChIP-seq) assay [24,25]. ChIP-seq analyses validated target genes such as *HMOX1* and glutamate-cysteine ligase modifier/catalytic subunit (*GCLM*/*GCLC*) involved in heme degradation and redox regulation, respectively [24]. Further analyses of ChIP-seq data uncovered previously unidentified BACH1 binding regions involved in the functional pathways such as cell cycle, apoptosis, and cell proliferation. Another BACH1 ChIP-seq analysis using a primary mouse embryonic fibroblast (MEF) identified a novel BACH1 target gene, peroxisome proliferator-activated receptor gamma (*Pparg*), a key factor of adipogenesis related to lipid metabolism [25]. In addition, BACH1 ChIP-seq analyses suggested that BACH1 may have other binding partners other than MAF proteins when BACH1 bindings were compared with MAFK bindings using a MAFK ChIP-seq dataset [25]. BACH1 ChIP-seq showed that BACH1 mostly binds to the DNA element independent of MAFK protein, because bindings of BACH1 overlapped only about 11% with those of MAFK on the whole genome. Furthermore, noticeable differences were observed between BACH1 motifs and MAFK motifs identified in the ChIP-seq data set. However, it is not fully understood yet whether BACH1 has different partner proteins or binding cofactors to form oligomers or works as a monomer or homodimer for the transactivation of particular sets of target gene transcripts. It is noteworthy that BACH1 regulatory roles might be tissue-, cell-type-, or species-specific.

## 3. BACH1 Regulation in Cancer Cells

BACH1 expression is known to be regulated at post-transcriptional or translational levels in cancer cells. In breast cancer cells, a metastasis suppressor, Ras Kinase Inhibitory Protein (RKIP), indirectly suppresses BACH1 expression through a regulatory cascade that includes MAP Kinase, LIN28, and its downstream *let-7* miRNA [26,27]. It was revealed that BACH1 acts as a transcriptional suppressor of RKIP by binding on the upstream promoter of RKIP. Thus, RKIP and BACH1 create a mutually suppressive regulatory loop in breast cancer cells [8]. BACH1 promotes cancer metastasis and RKIP suppresses cancer metastasis, showing two molecules with opposite functions suppress each other. The mutually suppressive regulatory network between BACH1 and RKIP represents bi-stability, indicating that a tight balance between BACH1 and RKIP expression is needed to coordinate metastasis process of cancer cells. Additionally, BACH1 suppresses its own expression on the BACH1 promoter. Luciferase assays using upstream regions (+1130) from the transcription start site (TSS) of BACH1 showed reduced luciferase activity with BACH1 overexpression or increased luciferase activity with siRNA transfection for BACH1. BACH1 recruitment on the binding regions of the BACH1 promoter was validated through ChIP assays, indicating transcriptional regulation on its own expression. These suggest that cellular levels of BACH1 are also tightly regulated in human breast cancer cells through a negative feedback loop.

**Figure 2:**
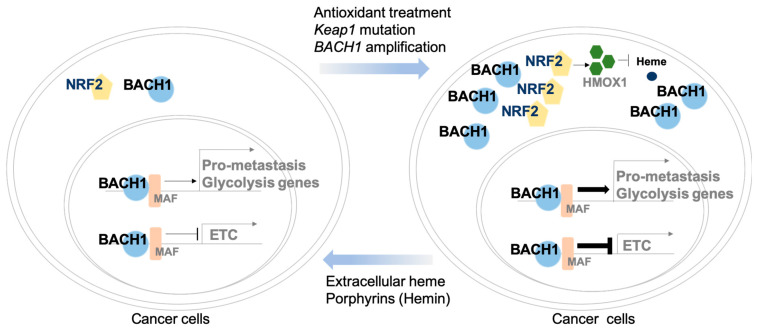
BACH1 regulates gene expression involved in cancer metabolism and cancer metastasis. BACH1 suppresses transcriptional activity of genes involved in mitochondrial electron transport chain (ETC), while BACH1 activates expression of genes involved in glycolysis including *HK2* and *GAPDH*, and pro-metastatic genes including *CXCR4* and *MMP1*. With Keap1 mutations or antioxidant treatment, BACH1 levels are more stabilized, thus BACH1 increases transcription of glycolysis genes and pro-metastatic genes, but decreases mitochondrial ETC genes. Exogeneous porphyrin treatment reduces BACH1 levels, modifying metastasis and metabolic programs of cancer cells.

Another regulatory mechanism of BACH1 expression includes mutation in either *Keap1* or *Nfe2l2* that comprises about 30% of human lung cancer [12]. In the *Keap1*-mutated lung adenocarcinoma, activated Nrf2 (encoded by Nfe2l2) stabilizes Bach1 expression that further promotes invasion and metastasis of lung tumors. Nrf2 is the master antioxidant transcriptional regulator against oxidative stress and is negatively regulated by Keap1, a Cul3-RING ubiquitin ligase, in the cell. Activated Nrf2 which is released from Keap1 mutation or Keap1 loss replaces BACH1 sites for transactivation of Hmox1 that stabilizes BACH1 in a Hmox1-dependent manner [21,22]. These findings clearly demonstrated that ubiquitin ligase Fbxo22 is involved in heme-dependent degradation of BACH1 [12]. Heme promotes the physical interaction between BACH1 and Fbxo22, identifying Fbxo22-interaction motifs with BACH1 through alanine scanning assays. When the residues at 9 phenylalanine (F9A), 11 tyrosine (Y11A), and 13 serine (S13A) of BACH1 were mutated, interaction of BACH1 with Fbxo22 was abolished. Furthermore, in vitro studies based on cell lines overexpressing wild type (WT) Bach1 or mutant Bach1 (Y11F) validated that mut Bach1 (Y11F) is more stable than WT Bach1 in abundant heme condition. Therefore, pharmacological inhibition of Hmox1 using Zn-PPIX resulted in the reduced levels of Bach1 expression through downregulated heme (Figure 2).

Furthermore, antioxidant treatment such as N-acetylcysteine (NAC) or vitamin E could stabilize Bach1 expression [11]. Lung cancer cell lines which were isolated and established from the Kras mutant mice with antioxidant treatment showed that antioxidant reduced reactive oxygen species (ROS) levels and stabilized Bach1 protein levels through heme, while BACH1 transcripts were not altered.

For the post-transcriptional gene expression regulation, *let-7* miRNA is known to suppress BACH1 levels by interacting with the 3′ untranslated region (UTR) of BACH1 in breast cancer cells and hepatocytes, [26,28,29]. MicroRNA (miRNA) is a small non-coding RNA fragment consisting of about 22 nucleotides for negative regulation of genes by interacting with the 3′ UTR of target genes [30]. BACH1 is also regulated by miRNA-155 or miRNA-330 for proliferation and migration of renal cancer cells or colon cancer [31,32]. Likewise, Bach1 is regulated by numerous factors in non-tumor tissues. Hypoxia-inducing-miR-532-5p also interacts with 3′ UTR of BACH1 to regulate muscular pericyte function including endothelial permeability, vascular stability, and angiogenesis [33]. In lung development, Bach1 expression was regulated by miRNA-196a expression [34]. Transient Bach1 induction was also observed in acute liver injury [35]. Hepatic injury by carbon tetrachloride (CCl_4_) demonstrated significant induction of Bach1 mRNA levels in rat animal models. Notably, hypoxia is known to induce *BACH1* transcripts, although it is unclear whether *BACH1* mRNA induction is mediated by HIFs [18].

## 4. BACH1 Regulates Cancer Metastasis

BACH1 is expressed aberrantly higher in breast tumor than non-tumor control tissues [36]. In particular, basal-like breast cancer or triple-negative breast cancer (TNBC) subtype displays distinctive levels of *BACH1* transcripts when compared with other breast cancer subtypes such as luminal A, luminal B, or her2-positive subtypes [10,36]. *BACH1* expression in tumors are mainly due to genetic amplification as validated in breast cancer or increased protein stability as validated in lung cancer [11,12]. BACH1-enriched cancer cells showed promoted metastatic properties such as migration, invasion, intravasation and metastasis of cancer cells both in vivo and in vitro [26,27,37]. In breast cancer cells, BACH1 enhanced expression of matrix metalloproteinases (*MMPs)* and CXC-chemokine receptor 4 (*CXCR4*) mRNA as a transcriptional activator through direct interaction on the promoter regions of target genes to promote metastatic progression, without affecting primary breast tumor growth (Figure 2) [26,37]. Also, a gene signature that consists of *BACH1* and its target genes including *MMP1* and *CXCR4* gives a worse prognosis for patients with TNBC indicated by shorter metastasis-free survival (MFS) rates within 5 years when analyzed in patient data cohorts (*n* = 878 and *n* = 470) [36]. Therefore, BACH1 suppression reduces metastatic processes such as migration, invasion, and metastasis [11,12,26,37,38].

Either antioxidant treatment-induced BACH1 or Keap1 loss-induced Bach1 promoted lung cancer metastasis [11,12]. Inhibition of Bach1 target gene, HMOX1, or Bach1-ligase overexpression could reduce Bach1-driven metastasis of lung cancer. Likewise, blockade of Bach1 target genes involved in the glycolysis pathways significantly reduced metastasis phenotypes in lung cancer. Moreover, BACH1 silencing showed decreased metastasis of pancreas cancer cells, both in vitro and in vivo, by inducing expression of a group of genes involved in epithelial-to-mesenchymal transition (EMT) process [38].

In addition to transcriptional regulation, BACH1 is also involved in epigenetic regulation of cancer cells [39,40]. In BRAF (V600E) mutant colon and skin cancer, BACH1 forms a complex with MAFG to mediate the function of DNA methyltransferase (DNMT3B) for hypermethylation of their target promoters to induce cancer progression.

Consistently, a transcriptional signature that includes Bach1 and its target genes showed strong association with poor survival, advanced clinical stage and grade, and metastasis in lung cancer patients [12]. Expectedly, BACH1 expression showed a positive correlation with poor prognosis in patients with kidney clear carcinoma or pancreatic adenocarcinoma, as well as with lung tumors [11]. Therefore, targeting BACH1 in cancer to suppress its levels might be beneficial to the cancer patients since BACH1 suppression could decrease tumor metastasis and increase metastasis-free survival of cancer patients. These suggest that BACH1 is a prospective target to treat metastatic tumors.

## 5. Function Roles of BACH1 in the Regulation of Metabolic Pathways in Cancer Cells

### 5.1. BACH1 Regulates Mitochondrial Metabolism of Cancer

Mitochondrial metabolism provides essential processes for energy metabolism, redox balance, and macromolecule biosynthesis in cancer cells [4]. A large amount of information indicates that intact mitochondria and active mitochondrial metabolism is pivotal for cancer cell survival and proliferation, thus it is suitable to be targeted for anticancer therapeutics [41]. Regulatory mechanisms of mitochondrial metabolism in cancer contain oncogenic signaling and mutated tumor suppressors, while some mitochondrial metabolites are also oncogenic [4,42].

Metabolomics, microarray and deep-sequencing approaches revealed that a metastasis promoting transcriptional factor, BACH1, participates in metabolic reprogramming of cancer cells [10,27]. Metabolic regulatory function of BACH1 in mouse embryonic fibroblasts (MEFs) was feasibly suggested by Bach1 ChIP-seq analyses [25]. Lipid metabolism such as adipogenesis was detected as a BACH1 target, since peroxisome proliferator-activated receptor gamma (*PPARg*) was observed as a binding target gene of BACH1 in the ChIP-seq results.

**Figure 3:**
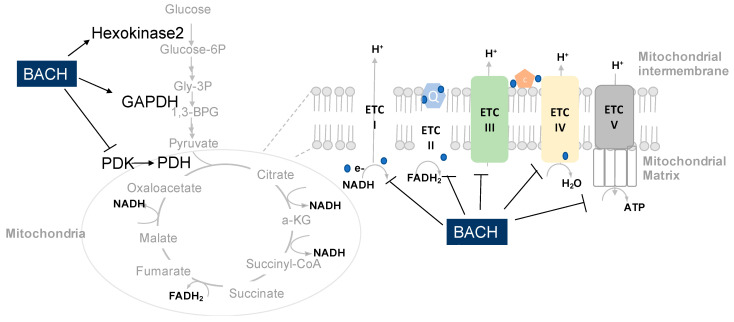
BACH1 regulates key genes involved in central carbon metabolic pathways of cancer cells. BACH1 enhances glycolysis pathway by activating expression of *HK2* and *GAPDH* mRNA, while it suppresses mitochondrial metabolism by inhibiting PDH activity through *PDK* regulation and expression of the subunit’s genes of mitochondrial ETC complex (I–V) that are coupled to the TCA cycle. (Abbreviations: Gly-3P; glyceraldehyde 3-phosphate, 1,3-BPG; 1, 3-bisphosphoglycerate, PEP; phosphoenol pyruvate, PDK; pyruvate dehydrogenase kinase, PDH; pyruvate dehydrogenase, aKG; alpha ketoglutarate, HK2; hexokinase, GAPDH; glyceraldehyde 3-phospho dehydrogenase, ETC; electron transport chain, TCA cycle; the citric acid cycle, Q: ubiquinone, C; cytochrome *c*, ATP; adenosine triphosphate, NADH; nicotinamide adenine dinucleotide, FADH_2_; flavin adenine dinucleotide 2).

In TNBC cells, mitochondrial inner membrane genes are the most significantly changed targets upon BACH1 depletion using shRNA [10]. As a transcriptional factor, BACH1 inhibits transcriptional expression of genes involved in mitochondrial oxidative phosphorylation (OXPHOS) of breast cancer cells as observed by reverse-transcriptase quantitative PCR (qRT-PCR) and protein blotting (Figure 3). In support of these findings, bioinformatic approaches using KEGG annotation assays presented that mitochondrial OXPHOS is the most inversely associated functional pathway with BACH1 expression in patient tumors including breast, skin, liver, pancreas, and prostate. BACH1 supports aerobic glycolysis by inhibition of mitochondrial electron transport chains (ETC) gene expression in breast tumors. When BACH1 is reduced, mitochondrial ETC genes are de-repressed, thus enhancing mitochondrial respiration as assessed by increasing oxygen consumption rates (OCR) while decreasing extracellular acidification rates (ECAR) of TNBC cells [10]. In addition, the activity of pyruvate dehydrogenase (PDH) that converts pyruvate to acetyl CoA for the entry of the tricarboxylic acid (TCA) cycle was also suppressed by BACH1 through pyruvate dehydrogenase kinase (PDK) regulation. Furthermore, flux analyses using ^13^C-labeled glucose showed that BACH1 suppresses incorporation of glucose into the TCA cycle intermediates, as metabolomics analyses validated substantially higher levels of glycolysis intermediates and lower levels of the TCA cycle intermediates in the BACH1-enriched TNBC cells relative to the BACH1-depleted cells.

Since BACH1 regulates the central carbon metabolism in TNBC cells, reducing BACH1 levels either genetically or pharmacologically could rewire metabolic pathways, by enhancing mitochondrial respiration and reducing aerobic glycolysis [10]. The flexibly altered cancer metabolism by BACH1 inhibition also plays as a primary metabolic pathway in cancer, thereby generating metabolic liability when this primary metabolism is blocked. Subsequently, mitochondrial respiratory inhibitors such as metformin, rotenone, and antimycin A could be even more lethal to the survival of cancer cells that are depleted with BACH1 compared to the BACH1-enriched control [10]. Additionally, transient silencing of *COX15* or *UQCRC1* using siRNA in the BACH1-depleted cells as low as the levels of those in control cells completely restored metformin resistance and rescued cell growth. These results indicate that mitochondrial OXPHOS plays a critical role for metformin resistance in TNBC cells and is altered by BACH1 manipulation. Notably, BACH1 does not alter the expression of metformin transporter, OCT1 (encoded by *SLC22A1*), indicating that the metformin transporter is not involved in the metformin sensitivity by BACH1 in TNBC cells [10]. Moreover, mitochondrial biogenesis genes such as PPARg or peroxisome proliferator-activated receptor gamma coactivator1-alpha (PGC1a, encoded by *PPARGC1A*) was not changed by BACH1 in TNBC cells. These data suggest that BACH1 regulates mitochondrial metabolism independent of PGC1a in TNBC cells.

However, addition of pyruvate into the growth media of BACH1-depleted cells rendered drug resistance to metformin treatment [10]. The TNBC cells that are enriched with BACH1 are resistant to metformin treatment regardless of pyruvate, but shBACH1 cells were only resistant to metformin in the presence of pyruvate. The effect of pyruvate was seen in the changes of NAD^+^/NADH ratio as previously reported [43]. NAD^+^/NADH ratios are important factors in determining metformin responses of cancer cells and were also altered by BACH1. In addition, preclinical studies and bioinformatic approaches suggest that breast tumors expressing low levels of BACH1 might be potentially sensitive to metformin treatment, while those enriched with BACH1 might be resistant to metformin treatment when administered as a single agent to manage tumors [10]. These findings suggest that combining metformin with hemin (or any other possible BACH1 inhibitor) would effectively suppress growth of tumors from patients who did not respond to metformin for tumor suppression due to their enriched high BACH1 levels.

In further studies, metformin treatment suppressed tumor growth from the mice xenografted using shBACH1 cells relative to the BACH1-enriched control tumors [10]. Moreover, mice xenografted with BACH1-enriched breast cancer cells or TNBC patient-derived xenograft (PDX) mice showed significantly tumor suppression when mice were injected with hemin to reduce BACH1 levels and metformin. In contrast, mouse models bearing mutant Bach1-expressing TNBC tumors did not suppress tumor size when both metformin and hemin were administered, because hemin did not interact with mutant Bach1. These data indicate that targeting BACH1 increases response of metformin treatment, suggesting a novel combination therapeutic strategy using hemin and metformin. In support of preclinical studies, bioinformatic analyses of patient tumor expression data from The Cancer Genome Atlas (TCGA) highlighted mitochondrial OXPHOS regulated by BACH1 in numerous cancer types. Altogether, BACH1 may play a conserved role for mitochondrial metabolism in diverse cancer types. The flexible metabolic phenotypes of cancer that are altered by BACH1 suggest BACH1 as a useful target for cancer vulnerability against mitochondrial inhibitors [41].

### 5.2. BACH1 Regulates Redox Stress of Cancer

Redox regulation is another key physiological function of cells for cell survival against oxidative stresses. BACH1 and NRF2 are well known to interplay for cellular redox homeostasis [18]. BACH1 suppresses antioxidant genes by binding to the Antioxidant Response Element (ARE) of antioxidant genes, while NRF2 activates expression of antioxidant genes on the same sites upon receiving stress signals. Under oxidative stress, cells induce free heme by releasing them from heme-containing proteins, which further degrade Bach1 to de-repress antioxidant gene expressions that are suppressed by Bach1 [23]. In this mechanism, oxidative stress accumulates NRF2 proteins for transactivation of antioxidant genes including HMOX1 that further alleviate free heme levels to rescue BACH1 protein levels back, and not BACH1 transcripts. The counteractions between BACH1 and NRF2 play a critical role for managing cellular stresses by turning on and off antioxidant gene expression. Therefore, extracellular or intracellular interruption of the BACH1 and NRF2 regulatory loop could be detrimental for cells burdening reactive oxygen species (ROS) stress or ROS-mediated signaling for tumorigenesis [11,12]. One example is that exogenous antioxidant treatment such as N-acetyl cysteine (NAC) or vitamin E in a water-soluble could promote metastasis process of KRas-mutated lung cancer cells, through reducing intracellular free heme that increases BACH1 proteins levels [11]. Stabilized BACH1 as a major driver facilitated metastasis of lung cancer cells. Pharmacological inhibition of HMOX1 using ZnPPIX was able to suppress lung cancer metastasis by inducing Bach1-destabilization in a Fbxo22-dependent manner [12].

ROS is a consequential product of hyperactivated mitochondrial respiration, which was observed higher in shBACH1 TNBC cells than controls [10,41]. Induced ROS levels by BACH1 depletion was not sufficient to affect either survival or death of TNBC cells, because BACH1 depletion, as a defense mechanism, could de-repress expression of glutathione synthesis genes to counterbalance increased ROS. It has been shown that BACH1 depletion induces expression of glutathione (GSH) biosynthesis such as glutamate-cysteine ligase modifier (*Gclm*) and catalytic subunit (*Gclc*), and cystine/glutamate antiporter xCT, encoded by solute carrier family 7 member 11 (*Slc7A11*) [44].

Based on the fact that glutathione is involved in the inhibition of ferroptosis, which is a type of programmed cell death triggered by iron, BACH1 also showed regulatory function of ferroptosis [44]. Combined analyses of ChIP-seq and RNA-sequencing using mouse myeloblast M1 cells identified Bach1-target genes including ferritin genes (*Fth1* and *Tfl1*) and ferroporin (encoded by solute carrier family 40 member 1, *Slc40a1*) by binding to the regulatory region of genes. Further, expression of Bach1 target genes were validated using qPCR and Western blotting in the Bach1^-/-^ MEFs, indicating BACH1 involvement in lipoperoxidation and iron metabolism. Therefore, ferroptosis promoted by BACH1 could impair severity of diseases such as acute myocardial infarction [44].

### 5.3. BACH1 Regulates Glycolysis of Cancer Cells

Aerobic glycolysis in the presence of oxygen represents convergent cancer metabolic phenotypes, providing a diagnostic foundation for tumor detection [1,2,4,42], and this aerobic glycolysis is regulated by BACH1 in lung cancer cells [11]. Integrated analyses of RNA-seq and ChIP-seq to access BACH1 targets using lung adenocarcinoma identified that BACH1 activates transcription of hexokinase 2 (*HK2*) and Glyceraldehyde 3-phosphate dehydrogenase (*GAPDH*) at their promoters as a transcriptional activator (Figure 3) [11]. Although *HK2* mRNA was the most significantly upregulated gene in the cells after treatment with antioxidants, hexokinase 1 (*HK1*) was not changed. In addition, transcripts of 6-phosphofructo-2-kinase/fructose-2,6-bisphosphatase 3 (*PFKFB3*) and solute-carrier family 16 member 1 (*SLC16A1*) were increased in cells treated with antioxidants, suggesting a positive correlation with BACH1 expression in patient tumors.

Antioxidant-treated lung cancer cells showed higher glycolysis rates than control cells that are assessed by ECAR, OCR, increased glucose consumption, and increased lactate production in a BACH1-dependent manner [11]. Furthermore, antioxidant treatment increased metastasis of lung cancer cells through activated glycolysis. Enhanced ATP levels from the activated glycolysis in the antioxidant-treated cells became an energy source to support increased invasiveness and metastasis in a Bach1-dependent manner. Activation of glycolysis pathway by exogenous expression of HK2 could induce invasiveness and metastasis of control lung cancer cells. However, knockout of Bach1 using single-guide RNA (sgBach1) normalized ATP levels, invasiveness, and metastasis of antioxidant-treated cancer cells to the levels of control cells. Inhibition of glycolysis pathways, therefore, was effective to suppress cancer metastasis in both antioxidant-treated and Bach1-induced lung cancer models. Small inhibitory molecule treatment such as 2-deoxyglucose (2-DG) and lonidamine for inhibition of HK2, 3-bromopyruvate (3-BP) for inhibition of GAPDH, dichloroacetate (DCA) for inhibition of PDK, and AZD3965 for inhibition of monocarboxylate transporter (MCT1, encoded by *SLC16A1*) resulted in reduced migration or metastasis of antioxidant-treated lung cancer cells. Whereas, inhibition of mitochondrial pyruvate carrier (MPC) using UK5099 or inhibition of pentose phosphate pathway (PPP) using 6-aminonicotinamide (6-AN) did not change migration of either antioxidant-treated or control lung cancer cells [11]. Taken all, BACH1 mediates activation of aerobic glycolysis pathways and the metastatic process of lung cancer cells, as inhibition of BACH1 using either shRNA, sgRNA, or hemin treatment decreases migration and metastasis of cancer cells through aerobic glycolysis reduction.

## 6. Pharmacological Inhibition of BACH1

The heme regulatory role of BACH1 supports that BACH1 protein can be leveraged through excessive free heme (or hemin), suggesting hemin as a pharmacological tool. Hemin (Haemin) is an iron-containing porphyrin (Iron-protoporphyrin IX) that is from a heme group [45]. As an essential cofactor in cellular processes, hemin plays important roles for numerous biological functions including oxidative stress response. Injection of hemin is currently in a form of drug (Panhematin), which is approved by the Food and Drug Administration (FDA) to treat patients with acute porphyria [46]. In a previous report, hemin treatment to breast cancer cells and the mice bearing breast tumors exhibited substantially reduced BACH1 levels with unnoticeable toxicity, suggesting hemin as a potentially non-toxic BACH1-specific drug for tumors [10]. Hemin treatment significantly decreased BACH1 levels in tumors and altered metabolic pathways which are downstream of BACH1. Also, hemin treatment increased sensitivity of cancer cells against mitochondrial inhibitors including metformin, a diabetic drug, both in vivo and in vitro tumor models. Importantly, hemin specificity for BACH1 as a BACH1 drug was tested using a heme-resistant murine mutant (mut) Bach1 (mut Bach1), which has cysteine (C) to alanine (A) point mutations in four of the heme-binding motifs that are required for heme binding. Thus, mut Bach1 is resistant to heme binding, release of BACH1 from DNA for nuclear export, and subsequent degradation of BACH1 upon excessive heme treatment [10,21,43]. Mut Bach1 in TNBC cells still contains BACH1 function for target gene regulation, metabolic pathway regulation, and drug sensitivity. TNBC cells expressing mut Bach1 did not respond to hemin treatment for Bach1 protein degradation, revealing hemin action as a BACH1 specific drug.

In addition to heme, cadmium is known to induce nuclear export of Bach1, enhancing expression of target genes including *HMOX1* in response to oxidative stress [47]. The CLS domain conserved at the C-terminal of Bach1 and Bach2 is responsible for cadmium response. Since cadmium activates p38 and ERK1/2 pathways for Nrf2 activity, Bach1 is also regulated by p38 and ERK1/2 signaling. However, Bach1 remained in the nucleus in the presence of cadmium when cells were treated with a MEK1/2 inhibitor. Thus, it has been suggested that cadmium may inactivate Bach1 through another mechanism in addition to nuclear export.

Moreover, a heme-related molecule, Fe^2+^ mesoporphyrin IX (FeMePIX), is able to interact with Bach1 to block DNA binding of Bach1, which further indicates FeMePIX as a potential cellular inhibitor of BACH1 for transactivation [21]. Likewise, zinc(II) mesoporphyrin and cobalt(II) porphyrin are known to contribute upregulation of *HMOX1* mRNA expression through BACH1 degradation in cells [48]. Additionally, small inhibitory molecule, HPP-4382, is suggested as a BACH1 modulator showing induced *HMOX1* mRNA levels through the suppression of BACH1 activity as validated by ChIP and reporter assays [49].

## 7. Targeting BACH1 is Clinically Beneficial

The functional role of BACH1 for cancer metabolism was not validated only by gene expression analysis of the patient data cohorts such as The Cancer Genome Atlas (TCGA) including breast, lung adenocarcinoma, and pancreas ductal adenocarcinoma, but by follow-up experiments [10,11,12,26,36]. Obviously, BACH1 serves as a diagnostic marker to stratify patient outcomes with numerous cancer types including breast and lung cancer. The gene signatures consisting of BACH1 and target genes are clinically useful to predict outcomes of patients with tumors.

**Figure 4:**
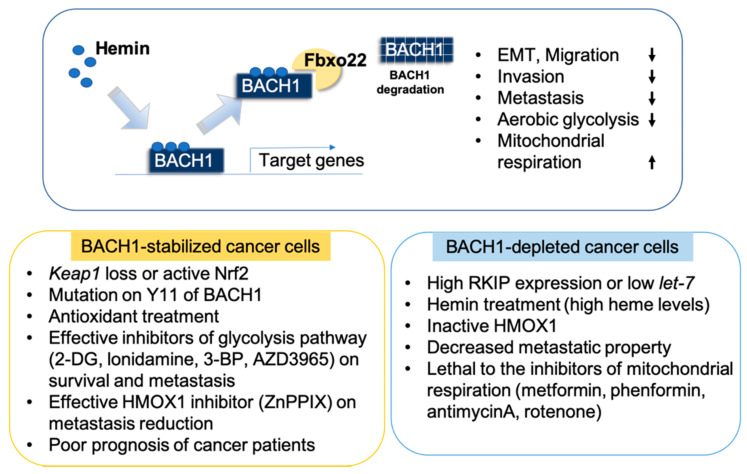
Pharmacological inhibition of BACH1 for cancer therapeutics. Alteration of BACH1 levels and cellular phenotypic changes by BACH1 levels are summarized. Hemin is a non-toxic BACH1 drug, causing degradation of BACH1 through ligase Fbxo22 interaction. BACH1 depletion modifies expression of target genes of BACH1 both positively and negatively at the transcription levels. BACH1 depletion decreases EMT, migration, invasiveness, metastasis, aerobic glycolysis, but increases mitochondrial oxidative phosphorylation and the coupled TCA cycle.

In particular, blockade of BACH1 using either approach including shRNA, sgRNA, or small inhibitors such as hemin, reduced BACH1 levels in cancer cells and reduction of BACH1 was sufficient to successfully and efficiently inhibit metastasis processes of cancer cells. Importantly, blockade of BACH1 rewires metabolic liability, which further generates metabolic vulnerability that can be targeted with metabolic inhibitors as a novel combinatory therapeutics. Furthermore, inhibiting BACH1 downstream targets such as mitochondrial metabolic pathways, glycolysis pathways, heme regulatory pathways, and redox homeostasis might be a highly insightful approach to manage metastatic cancer in a complex and context-dependent tumor microenvironment (Figure 4).

## 8. Conclusions

Distinct metabolic pathways that support rapid tumor growth became a crucial resource to achieve effective treatments of cancer as well as to identify accurate prognostics and cancer diagnostics in clinics. Currently, numerous drugs that inhibit metabolic pathways, including nucleic acid biosynthesis, folate metabolism, and amino acid metabolism have been approved for cancer treatment and widely used for many cancers. Above all, plasticity of cancer metabolism generates ineffectiveness of drugs that inhibit metabolic pathways, unless cancer metabolism is rewired to be targetable. Therefore, understanding cancer-specific metabolism and its molecular mechanisms will provide information to restrict metabolic plasticity of cancer cells so as to obtain effective therapeutic intervention targeting key metabolisms.

In conclusion, BACH1 is a useful therapeutic target for metastatic cancer, as highlighted by recent publications. BACH1 regulates primary metabolic processes such as aerobic glycolysis, mitochondrial OXPHOS, the TCA cycle and redox regulation of cancer cells. Inhibition of BACH1 could be achieved by small molecules including hemin. Hemin is well validated as a BACH1-specific non-toxic drug with its broad application. Particularly, targeting BACH1 using hemin when combined with a metabolic inhibitor, metformin, projected promising therapeutic intervention as a novel combination drug for cancer patients. While recent studies identified new roles of BACH1 in the primary cancer metabolism, there are still other metabolic pathways regulated by BACH1 to understand, including: one carbon metabolism, amino acid metabolism, pentose phosphate pathway, and fatty acid oxidation. As briefly mentioned earlier, BACH1 plays a role for macrophage metabolism, which might be critical for communication between cancer and tumor-infiltrated macrophages. In future studies, it needs to be explored for understanding of metabolic communication between cancer cells and immune cells including macrophages infiltrated into tumors [50].
